# DGAT1 and ABCG2 polymorphism in Indian cattle (*Bos indicus*) and buffalo (*Bubalus bubalis*) breeds

**DOI:** 10.1186/1746-6148-2-32

**Published:** 2006-11-07

**Authors:** Madhu S Tantia, Ramesh K Vijh, Bishnu P Mishra, Bina Mishra, ST Bharani Kumar, Monika Sodhi

**Affiliations:** 1 National Bureau of Animal Genetic Resources, Karnal 132001, India

## Abstract

**Background:**

Indian cattle (*Bos indicus*) and riverine buffalo (*Bubalus bubalis*) give a poor yield of milk but it has a high fat and protein percentage compared to taurine cattle. The identification of QTLs (Quantitative Trait Loci) on BTA14 and BTA6 and its subsequent fine mapping has led to identification of two non conservative mutations affecting milk production and composition. Our objective was to estimate the frequency of K232A (DGAT1 – diacylglycerol – acyltransferase 1) and Y581S (ABCG2 – ATP binding cassette sub family G member 2) polymorphisms in diverse cattle and buffalo breeds of India having large variation in terms of milk production.

**Results:**

We screened the reported missense mutations in six cattle and five buffalo breeds. The DGAT1^K ^and ABCG2^Y ^alleles were found to be fixed in Indian cattle and buffalo breeds studied.

**Conclusion:**

This study provides an indirect evidence that all the Indian cattle and buffalo breeds have fixed alleles with respect to DGAT1 and ABCG2 genes reported to be responsible for higher milk fat yield, higher fat and protein percent.

## Background

Significant development and refinement of analytical tools has resulted in identification of causal genes underlying QTLs (Quantitative Trait Loci) for various economic traits. is The detection of sequence variations of causal genes affecting the traits of interest are now possible in humans, animals and plants [1].

Several studies in cattle [2-4] identified a QTL for milk production traits especially the milk fat percentage on chromosome 14 (BTA 14) near the centromeric region. A comparative positional candidate gene approach led to identification of the candidate gene coding acylcoA: diacylglycerol – acyltransferase 1 (DGAT1). DGAT1 is considered to be the key enzyme in controlling the synthesis rate of triglycerides in adipocytes. Smith et al. [5] demonstrated absence of lactation in knockout mice lacking both copies of DGAT1. Grisart et al. [6,7], Winter et al. [8] and Weller et al. [9] identified a polymorphism in exon 8 of the DGAT1 gene in *Bos taurus*, AA → GC exchange resulting in a non conservative substitution of amino acid 232 Lysine (K) → Alanine (A). This polymorphism has been associated with increased fat yield, fat and protein percent as well as decrease in milk and protein.

Another segregating QTL for milk production trait on BTA 6 was found by many workers in various populations of *Bos taurus *[10-14]. The QTL centered around microsatellite BM143. Olsen et al. [15] used physical mapping and combined linkage and linkage disequilibrium mapping to fine map QTL region between ABCG2 and LAP3. Several genes like PKD2 [15], SPP1 (Osteopontin) gene [16,17] and ABCG2 (ATP binding cassette sub family G member 2)[18] have been proposed as candidate genes in the QTL region. Cohen-Zinder et al. [18] analysed the sequence variation of these genes and demonstrated that ABCG2 (Y581S) allele (SNP A → C in exon 14) to be the only polymorphism which corresponded to the segregation status of the heterozygous and homozygous sires for the QTL based on the allele substitution effect.

These two QTNs (Quantitative Trait Nucleotide) DGAT1^K ^and ABCG2^Y ^are proposed non conservative mutations in *Bos taurus *associated with increased fat yield, fat and protein percent as well as decrease in milk yield. A study was carried out to adjudge the status of these mutations in *Bos indicus *and *Bubalus bubalis*.

## Results and discussion

**Figure 1:**
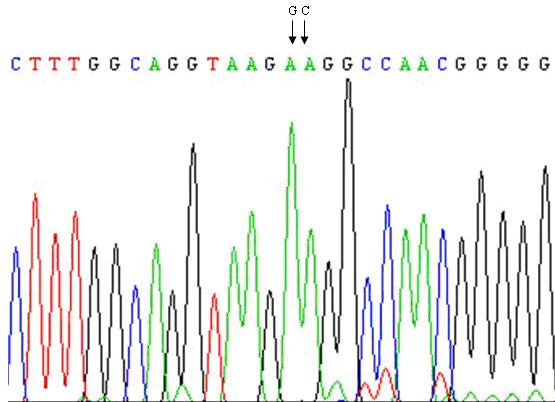
Chromatograph of DGAT1 gene indicating AA → GC (K232A) mutation in Exon8.

The sequence obtained for segment of DGAT1 in cattle (DQ228762) and buffalo (DQ228763); have been submitted to NCBI database. The sequence analysis of DGAT1 region in six breeds of cattle (*Bos indicus*) and 5 breeds of buffalo (*Bubalus bubalis*) revealed fixed DGAT1^K ^allele (Fig. 1). The sequence of desired region of control individuals revealed heterozygous condition for reported mutation in 3 animals. No variation was observed in all the 11 breeds of cattle and buffalo undertaken in this study. Kaupe et al, [19] had reported fixed DGAT1^K ^allele in Nellore cattle. Lacorta et al [20] also reported absence of A allele in Nellore and Guzerat cattle and very low frequency (<5%) in Gyr and Redsindhi cattle of Brazil. High frequency of DGAT1^A ^has recently been reported in Uruguayan Creole cattle [21] and Holstein and its crosses [20]. This polymorphism has been associated with increased fat yield, fat and protein percent as well as decrease in milk and protein production.

**Figure 2:**
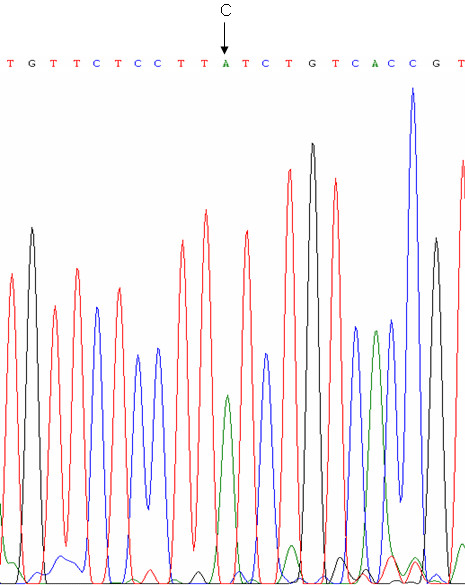
Chromatograph of ABCG2 gene indicating A → C (Y581S) mutation in Exon14.

The sequence obtained for ABCG2 (DQ205445 for cattle and DQ205444 for buffalo) have been submitted to NCBI database. The analysis of sequence of ABCG2 region in Indian breeds of cattle (*Bos indicus*) and buffalo (*Bubalus bubalis*) revealed fixed ABCG2^Y ^allele (Fig. 2). Only one animal of control group was heterozygous for ABCG2 allele. Cohen-Zinder et al, [18] reported increase in frequency of ABCG2^Y ^allele with selction for higher milk fat and protein percentage in the Israeli Holstein population.

## Conclusion

This study provides an indirect evidence that all the Indian cattle and buffalo breeds have fixed alleles with respect to DGAT1 and ABCG2 genes reported to be responsible for higher milk fat yield, higher fat and protein percent. There is considerable genetic variation in milk production in India which can be exploited to increase the milk yield without having an adverse effect on fat and protein percent.

## Methods

The DNA samples of 20 unrelated animals from Sahiwal, Rathi, Deoni, Tharparkar, Red Kandhari and Punganur breeds of cattle; and Murrah, Jaffarabadi, Surti, Mehsana and Bhadawari breeds of buffalo were isolated. The blood samples were collected over a large area in the native tract of breeds where AI services were not in operation to avoid any relationship among the sampled individuals to the extent possible. Five samples of *B. taurus *× *B. indicus *crosses were taken as control. We sequenced the DGAT1 and ABCG2 regions for known mutations by designing primers. The primer sequence for DGAT1 were: forward 5'-GCACCATCCTCTTCCTCAAG-3' and reverse 5'-GGAAGCGCTTTCGGATG-3'[19] and for ABCG2 were: forward 5'-CAGGGCTGTTGGTAAATCTCA-3' (nt 62491–511) and reverse 5'-GCACGGTGACAGATAAGGAGA-3' (nt 62580–600) from NCBI database sequence AJ871176

These PCR conditions were standardized using gradient PCR (iCycler, Bio-Rad Laboratories, Hercules, CA, USA). For DGAT1 PCR conditions were 5 min at 95°C; 35 cycles 45 s at 95°C, 45 s at 57°C, 45 s at 72°C; and a final 10 min extension at 72°C. For ABCG2 touch down PCR profile included 5 min at 95°C; 5 cycles 45 s at 95°C, 45 s at 57°C, 45 s at 72°C; 15 cycles 45 s at 95°C, 45 s at 56°C, 45 s at 72°C; 15 cycles 45 s at 95°C, 45 s at 55°C, 45 s at 72°C; and a final 10 min extension at 72°C. The PCR products were purified with Exo-AP (New England Biolabs, Beverely, MA, USA) digestion and sequenced with Big Dye terminator chemistry (Applied Biosystems, California, CA, USA) on ABI 3100 Avant sequencer. The sequence analysis was performed using SeqScape software ver. 2.0 (Applied Biosystems, California, CA, USA).

## Authors' contributions

MST and RKV conceived the study and prepared the manuscript, BPM and BM sequenced the genes, ST and MS collected the blood samples and isolated DNA. All the authors read and approved the final manuscript.
